# Optimization in long-term survival after multiple arterial grafting in coronary artery bypass: A systematic review and meta-analysis

**DOI:** 10.1177/02184923251399733

**Published:** 2025-12-03

**Authors:** Aqyl Hanif Abdillah, Agustian Sofian, Auzan Hakim Agustian, Azzahra Fadhilah, Annisa Fatharani

**Affiliations:** 1 Faculty of Medicine, 245680 Universitas Sumatera Utara, Medan, Indonesia; 2 Department of Thoracic and Cardiovascular Surgery, 704232 Pertamina Pusat Hospital, South Jakarta, Indonesia; 3 Faculty of Medicine, 95338 Universitas Indonesia, Jakarta, Indonesia; 4 Faculty of Medicine and Life Sciences, 405987 King's College London, London, UK

**Keywords:** CABG, conduit selection, multiple arterial grafting, single arterial grafting, survival

## Abstract

**Introduction:**

Surgical revascularization through coronary artery bypass is a widely accepted approach for treating diseases affecting multiple coronary vessels. While the standard approach uses a single arterial graft combined with vein grafts, using numerous arterial grafts may improve long-term outcomes. Although supported by observational data and guideline recommendations, the broader adoption of multiple arterial grafting has been limited due to a lack of definitive randomized trial evidence and uncertainties in specific patient subgroups.

**Methods:**

A systematic review and meta-analysis were conducted to compare long-term survival in patients receiving multiple versus single arterial grafts during coronary artery bypass surgery. Twenty-seven studies (including one randomized trial) involving more than one million patients were included. The primary outcome was long-term all-cause mortality. Hazard ratios with 95% confidence intervals were pooled using a random-effects model. Subgroup analyses were performed based on age, sex, diabetes status, graft conduit type, extent of arterial revascularization, and left ventricular function. Meta-regression examined the impact of patient characteristics.

**Results:**

Multiple arterial grafting was associated with a significant reduction in long-term mortality compared to single arterial grafting. The pooled hazard ratio indicated an approximate 20% relative reduction in mortality. This survival benefit was consistent across all evaluated subgroups. Meta-regression did not identify any patient characteristic that significantly altered the benefit of multiple arterial grafting. No significant publication bias was detected.

**Conclusion:**

Multiple arterial grafting is associated with improved long-term survival in coronary artery bypass surgery. These findings support the broader implementation of this strategy in suitable patients while emphasizing the need for individualized surgical decision-making.

## Introduction

Coronary artery disease (CAD) is the leading cause of ischemic heart disease and a major contributor to global mortality. Ischemic heart disease is projected to account for 14.2% of all deaths by 2030.^1,2^ Among the most effective treatments for multivessel CAD is coronary artery bypass grafting (CABG), which restores blood flow to the ischemic myocardium using vascular conduits to bypass occluded coronary arteries.^1,3^ The choice of graft conduit has a significant impact on CABG outcomes. In particular, using the LITA to graft the left anterior descending (LAD) artery yields superior long-term results compared to using a saphenous vein graft (SVG), and this practice has been the standard of care for decades.^4^ The LITA-to-LAD strategy has remained dominant for over 30 years due to its proven patency and survival benefit.

More recently, multiple-arterial grafting (MAG) has been proposed as a means to enhance CABG outcomes further. Multiple-arterial grafting involves using two or more arterial conduits (commonly bilateral internal thoracic arteries [BITAs] or a LITA plus a radial artery [RA]) instead of the traditional single arterial graft (LITA) combined with vein grafts. Numerous studies have suggested that MAG can reduce both short- and long-term mortality compared to the single arterial grafting (SAG) approach.^5^ Accordingly, several cardiovascular societies, including the ACC/AHA,^6^ STS,^7^ and ESC/EACTS,^8^ have incorporated recommendations for considering MAG in appropriate patients. An extreme form of this approach is total arterial revascularization (TAR), in which all grafts are arterial with no veins used. Total arterial revascularization is typically achieved with either BITA grafts or a LITA supplemented by one or more RA grafts. The rationale for MAG (and especially TAR) is the superior durability of arterial conduits, where studies have shown that both a second ITA (RITA) and the RA have significantly better long-term patency than SVGs.^9^

Despite these theoretical advantages, the optimal conduit strategy in CABG remains a matter of debate, particularly when the LITA cannot be used (e.g., if the LITA is damaged or contraindicated due to patient factors such as severe frailty, obesity, chronic obstructive pulmonary disease, or only intermediate-grade LAD stenosis).^2^ In practice, the adoption of MAG has not been widespread. One primary reason is that while observational studies consistently report outcome benefits with additional arterial grafts, these benefits have not yet been conclusively proven in randomized controlled trials (RCTs), mainly due to the practical challenges of conducting such trials.^10^ Surgeons have also been cautious because of ongoing debates about which patients truly benefit from MAG. Conflicting results have been reported for specific subgroups—for instance, in patients over 70 years old, in those with diabetes mellitus (DM), in cases of severely reduced left ventricular ejection fraction (EF), with different choices of the second arterial conduit, and whether or not complete arterial (TAR) revascularization is achieved.^11^ These uncertainties have contributed to variability in surgical practice and hesitancy to employ MAG uniformly.

To address these gaps in the literature, we conducted a systematic review and meta-analysis to evaluate the long-term survival impact of multiple arterial grafting compared to SAG in CABG. In addition to assessing the overall effect on mortality, we performed subgroup analyses to determine whether the survival benefit of MAG is consistent across various patient populations (stratified by age, gender, presence of diabetes, type of second arterial graft, extent of arterial revascularization, and left ventricular function). We also carried out meta-regression analyses to investigate whether any of these factors modify the effect of MAG on long-term survival. Through this comprehensive approach, we aimed to provide more definitive evidence regarding the utility of MAG in contemporary CABG practice.

## Methodology

### Eligibility criteria

We included studies based primarily on observational designs but did not strictly limit the analysis to this type. Randomized clinical trials and other observational study designs were also considered if they were appropriate to the study objective. The review was developed using the PICO framework to define eligibility and guide the selection of studies. Our primary purpose was to evaluate the impact of multiple arterial grafting (MAG) on long-term mortality in patients undergoing CABG. Additionally, we aimed to examine whether patient characteristics, such as age, sex, type of secondary conduit, type of revascularization, DM, and low EF, and influenced outcomes. The PICO framework for this study is summarized in Table 1.

**Table 1.** PICO strategy.

Aspects	Criteria	Elaboration
**P**opulation	Adult patients (≥18 years) with multivessel coronary artery disease (CAD) undergoing isolated CABG.	Patients diagnosed with multivessel CAD who were eligible for CABG, regardless of comorbid risk factors. Excluded: Those undergoing concomitant cardiac surgery, those with congenital heart disease, or whom secondary conduits other than the internal thoracic artery (ITA) or radial artery (RA) were used. Studies conducted before 1980 were excluded to minimize bias from outdated surgical techniques ^12^.
**I**ntervention	Multiple arterial grafting (MAG)	Use of >1 arterial conduit, with or without supplementary saphenous vein grafts (SVGs). MAG configurations could include combinations such as LITA + RITA (BITA) or LITA + RA, at the surgeon's discretion. The main conduit was typically placed to the left anterior descending (LAD) artery. Total Arterial Revascularization (TAR) is defined as a subset of MAG in which all grafts are arterial.
**C**omparison	Single arterial grafting (SAG)	Use of one arterial conduit only (limited to ITA), with or without additional SVGs. The arterial graft was primarily used for the LAD territory. No supplementary arterial grafts were included.
**O**utcome	Primary Endpoint: Long-term Mortality	All-cause mortality at approximately 10 years post-surgery.

As outlined in the Population criteria, studies published before 1980 were excluded to reduce bias from outdated surgical techniques and to ensure that the included data reflect contemporary CABG practices. The period after 1980 was chosen because it marked pivotal advancements, including the widespread adoption of ITA grafting following evidence of its survival benefit,^13^ significant improvements in graft preservation methods, and the subsequent revival of RA grafting with modern antispasm protocols.^14^ These innovations fundamentally transformed surgical strategies and perioperative management after 1980, rendering earlier cohorts less comparable to current clinical practice.

To ensure consistency across included studies, we also applied strict definitions for the key grafting strategies analyzed. Multiple Arterial Grafting was defined as CABG procedures using two or more arterial conduits, regardless of whether supplementary SVGs were used. Total Arterial Revascularization was defined as a subset of MAG in which all conduits were arterial with no vein graft involvement. If a study used alternative terminology, its definitions were reviewed and harmonized with ours prior to inclusion. Additionally, to minimize confounding, we also prioritized studies that reported adjusted effect estimates, primarily using propensity score matching (PSM), inverse probability weighting (IPW), or multivariable regression techniques. These statistical methods were applied in most included studies to reduce baseline differences between the MAG and SAG groups.

### Literature searching method and systematic literature screening

We performed literature searching from September to October 2024 in specific databases as follows:

- PubMed;
- Cochrane;
- Google Scholar;
- ScienceDirect;
- Wiley Library.

We employed the PICO strategy to facilitate study selection and assess the suitability of the studies we encountered. The keywords applied for literature searching were expanded from the PICO strategy combined with Boolean Logic strategic searching method such as “CABG OR coronary artery bypass grafting,” “multiple arterial grafting OR MAG,” and “mortality” (((CABG) OR (coronary artery bypass)) AND (multiple arterial grafting) AND (mortality)) that were created based on the specification of each search engines or databases. We also manually screened the article's reference list for additional studies.

### Study selection

Our systematic review and meta-analysis study selection is based on the **P**referred **R**eporting **I**tems for **S**ystematic Reviews and **M**eta-**A**nalyses guidelines. The inclusion criteria for this study were the following:

1. Full-text article;
2. Published within the last 10 years;
3. Accessible;
4. English-written;
5. Reporting the long-term survival in MAG and SAG.

Meanwhile, this study's exclusion criteria are:

1. Systematic reviews, meta-analyses, literature reviews, case reports/series, editorials, commentaries, guidelines, books or book sections, conference abstracts, pilot studies, or study rationale/design papers;
2. Animal studies, in vivo/in vitro studies, or scientific statements/recommendations;
3. Retracted articles identified through publication databases.

For studies using shared registry data, we carefully evaluated overlapping patient cohorts to avoid duplication. If two or more studies were based on overlapping cohorts, only the primary publication was included in the main analysis to ensure accuracy and prevent bias. When the primary research did not meet the eligibility criteria or lacked sufficient data, a separate exploratory analysis from the same registry was used as an alternative. If different studies reported nonoverlapping data from the same registry, all relevant studies were included in the analysis. The screening and review process began with compiling all retrieved studies and removing duplicate records. Titles and abstracts were initially screened to identify potentially eligible articles, followed by a full-text review to confirm eligibility. Two authors independently reviewed each article to ensure objectivity. Inter-rater reliability was assessed using Fleiss’ kappa, which yielded a value of κ = 0.85, indicating almost perfect agreement among reviewers.^15^ Any further discrepancies and disagreements that arose between authors were resolved in discussion.

### Data extraction

The components of the studies extracted were as follows:

- *Participants:* Total number of participants of the study, number of participants enrolled, lost to follow-up and analyzed baseline characteristics, and inclusion and exclusion criteria.
- *Methods:* Design of the study, the duration of the study follow-up, study settings, and the date of the survey.
- *Interventions:* Intervention modalities and types, comparison, and cointerventions, if any.
- *Comparison:* The comparison modalities used in the subjects accounted for the control and the specified comparison of participants’ descriptions.
- *Outcomes:* Primary and additional endpoints of interest are specified in each study.
- *Additional highlights:* Limitations of the study and the study's conflicts of interest.

The data collected and extracted consisted of parameter amounts and specific units for each outcome, calculated using Microsoft Excel and R statistical software. The authors confirmed all of the data presented in this study to be available as part of the studies included within the published article or its supplementary materials.

### Risk of bias

Our meta-analysis comprises observational studies and RCTs. For observational studies, the risk of bias assessment was performed using the Risk of Bias in Non-Randomized Studies of Interventions tool, applying the guidance outlined in the Cochrane Handbook. For RCTs, the assessment was conducted using the Risk of Bias 2 tool, following advice in the Cochrane Handbook.

The quality of nonrandomized studies was assessed using the Newcastle–Ottawa Scale (NOS), whereas for RCTs, the assessment employed the Jadad scale. The evaluation was divided into three components: the selection, comparability, and outcome of the study. The final judgment of the study's quality was converted to Agency for Healthcare Research and Quality standards (good, fair, and poor). The total score range varies from 0 to 9 stars, in which studies with consequent results equal to or higher than 5 were considered to have adequate methodological quality to be analyzed.^16^ For the Jadad scale, the study is regarded as high quality if the total score is 3 or higher. The assessment of risk of bias is ultimately summarized in plots.

### Statistical design, data synthesis, and analyses

We employed various approaches to interpret our mathematical and structured analysis of this study, focusing on comparisons of specific outcomes represented by different parameters between the intervention group and the control group. The continuous data model was implemented to analyze both mean ± standard deviation and confidence interval (CI) values for study characteristics. The primary outcome was sought with ln(HR) and SE(ln(HR)) to obtain more accurate results from studies, and also used due to the nature of overall long-term mortality, which is an instantaneous occurrence, in contrast with risks, which are cumulative and generally found in several short-term outcomes after intervention. In addition, if the hazard ratio (HR) was not available in the published studies, we converted and sought the value using HR converting formulas from the available data served within the Kaplan–Meier curve introduced by Parmar et al. in 1998 to ensure the details of time-to-event analysis.^17^

Since heterogeneity across studies would be met, the meta-analyses were performed with inverse-variance analysis of the DerSimonian–Laird method within 95% CI, including all studies where they are considered suitable, appropriate, or similar enough in the eligibility criteria to be pooled. A value of *p* < 0.05 is considered statistically significant. Overall heterogeneity of the outcomes was concluded by the *I^2^* value, where < 30.0% represented “low heterogeneity,” and the values between 30.0% and 50.0% and >50.0% showed “moderate or some concern” and “high or substantial heterogeneity,” respectively.

The random effect size of the subsequent result, based on the analysis, was in our best interest for all the outcomes assessed. To minimize the potential bias in the assignments, only data from all matched studies were used, compiled through PSM, case–control matching, and IPW matching. On the other hand, RCT results were sought primarily from the intention-to-treat analysis, if available. Funnel plot construction illustrates the publication bias, which is used to prevent the tendency to show “only the significant” results in each outcome, and is statistically analyzed using both Egger's regression and Begg and Mazumdar's rank correlation tests.^18^ These analyses, along with meta-regressions, were performed with Microsoft Excel and R statistical software applications. Additionally, to reduce potential collinearity in the multivariable meta-regression, we limited the number of simultaneously included predictors and examined correlations among moderators such as age, diabetes, and low EF before model entry.

To prove and emphasize the preceding studies’ results regarding MAG benefits in specific populations, confirmatory subgroup analyses were performed with Bonferroni calculations for women, the elderly aged ≥ 70 years old, DM, and low EF.

## Results

**Figure 1.**
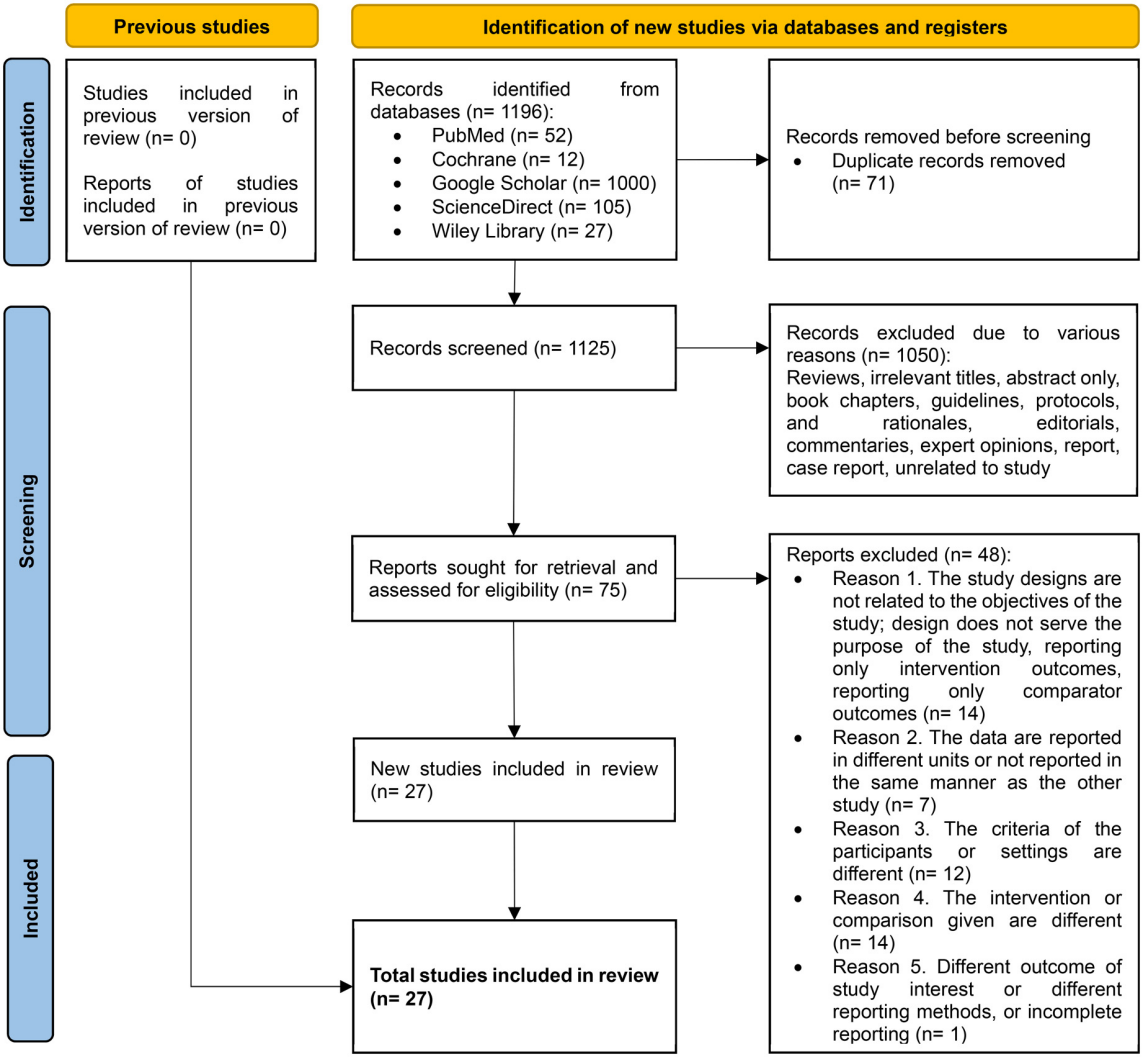
Preferred Reporting Items for Systematic Reviews and Meta-Analyses (PRISMA) chart of the study.

**Figure 2.**
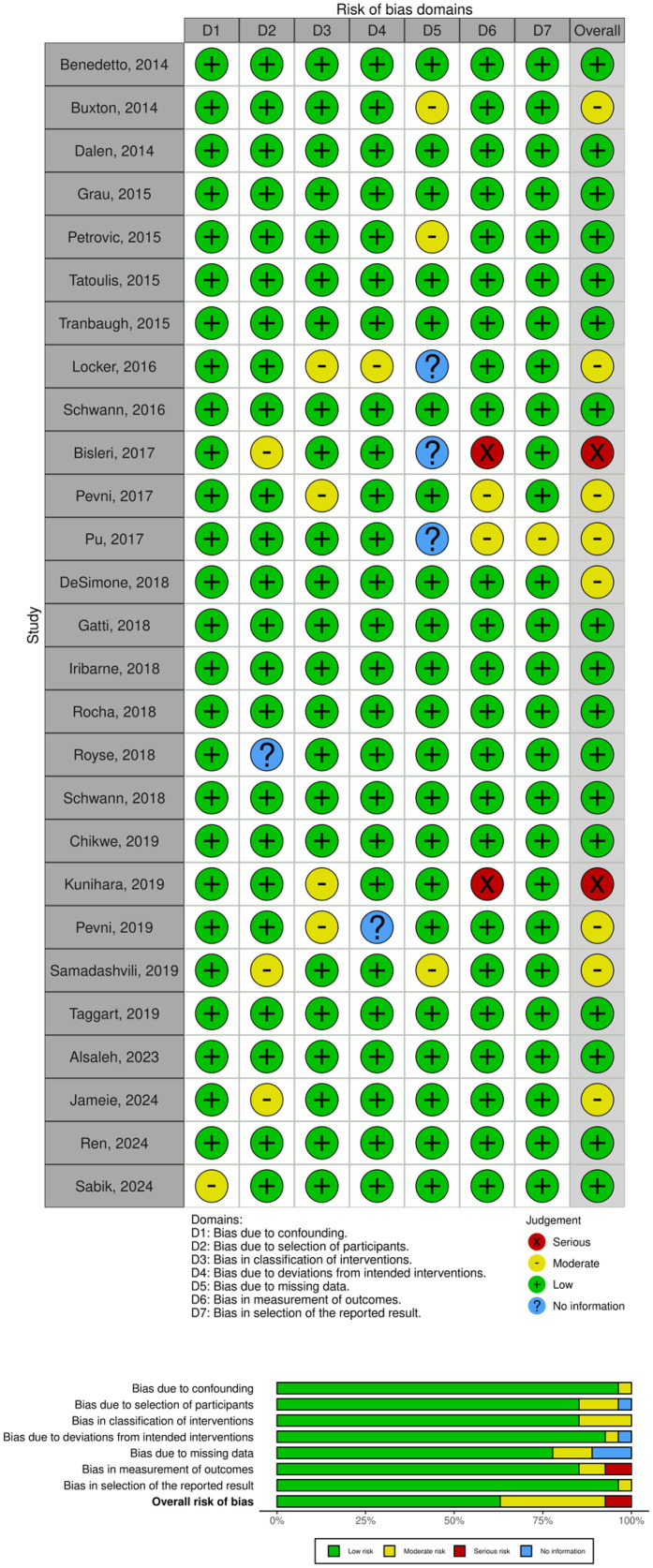
Risk of bias assessment of studies included using Risk of Bias in Non-Randomized Studies of Interventions (ROBINS-I) and Risk of Bias 2 (RoB 2) tool concluded in both traffic and summarized plot.

**Figure 3.**
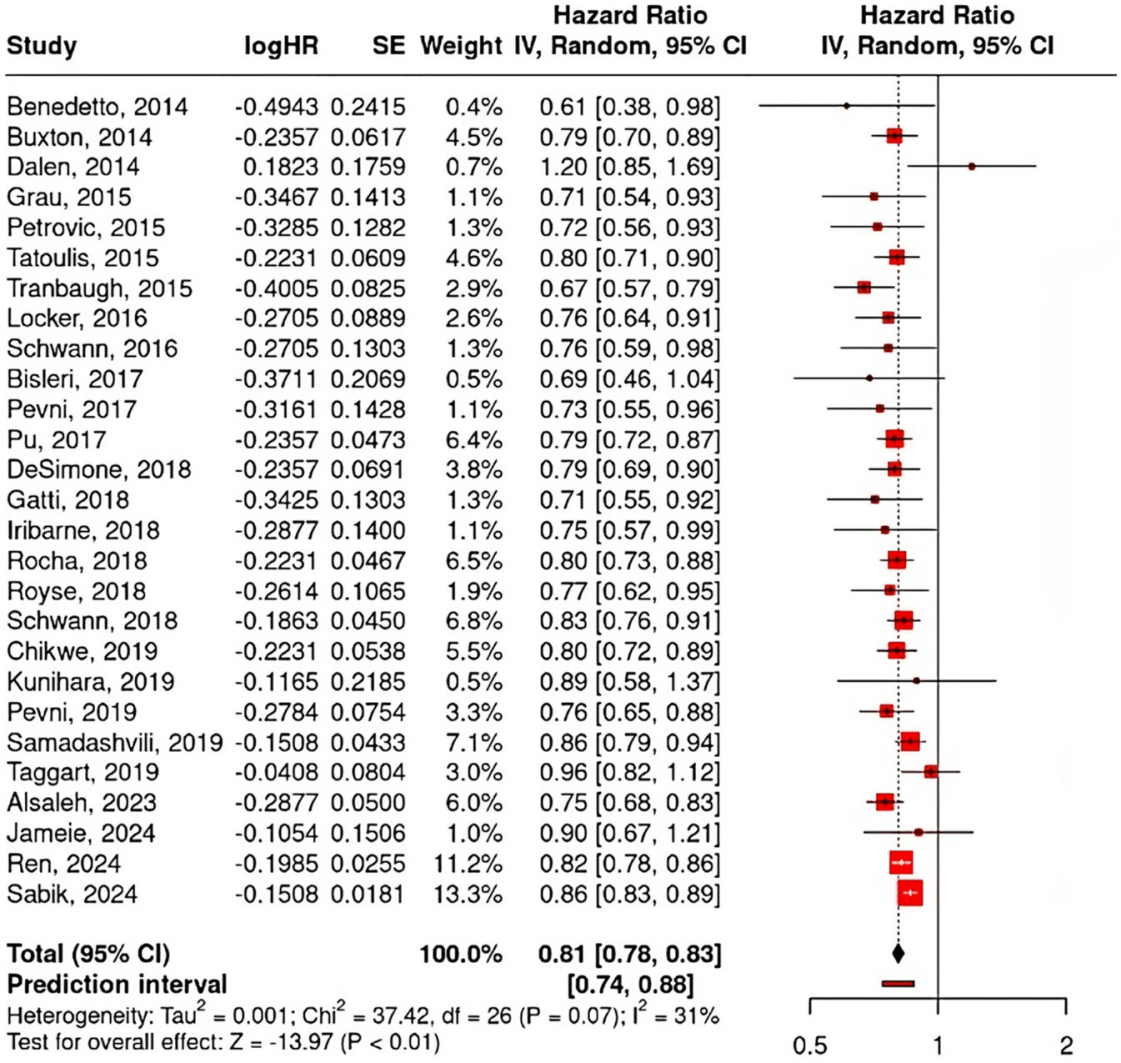
Forest plot of overall long-term mortality meta-analysis.

**Figure 4.**
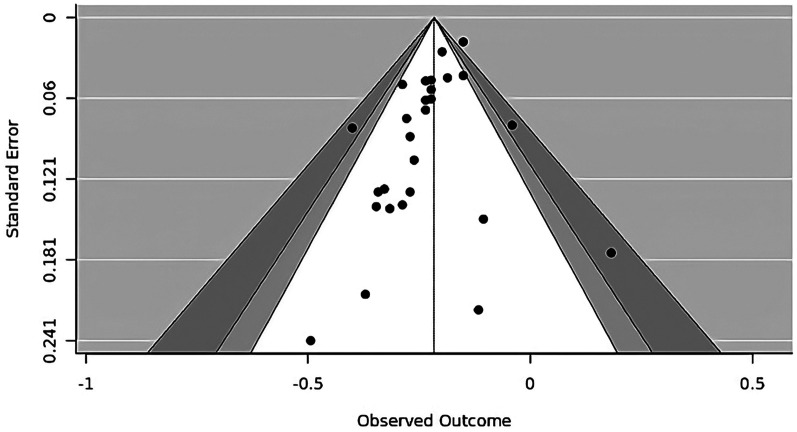
Funnel plot of long-term mortality meta-analysis.

A total of 1196 papers were obtained by literature searching in several search engines. After removing duplicates and screening titles and abstracts, 75 studies were selected for eligibility assessment and full-text retrieval. After reading the full text and assessing the study eligibility, we included a total of 27 studies, comprising matched-designed observational studies and RCTs as shown in Figure 1. The rest were excluded due to several reasons; 14 studies did not serve the purpose of our study, 7 studies’ data were not uniform or not reported in the reporting, 12 studies had different participants or settings, 14 studies did not provide suitable control or intervention, and 1 study was reporting a different outcome of interest, along with their method of report. The risk of bias for each study was assessed, as shown in Figure 2, along with the NOS and Jadad scales for evaluating study quality. The characteristics of each study are presented in Table 2. The meta-analysis and funnel plots are illustrated in Figures 3 and 4, respectively, whereas the meta-regression is summarized in Table 3.

**Table 2.** Characteristics of each study included.

Study and year	Country	Study type	Main second conduits of interest used	Participants, n intervention/control	Gender male, *n* (%) intervention/control	Age, mean ± SD	Comorbidities, *n* (%) intervention/control		Low ejection fraction (<50%), *n* (%) intervention/control	Off-pump CABG, *n* (%) intervention/control	Types of revascularization	Number of grafts used, mean ± SD	Clamp time, mean ± SD	CPB time, mean ± SD	Long-term mortality, HR (95% CI)	Long-term survival time of interest, in years	Mean or median follow-up time approximation, in years	NOS/Jadad scale
							DM	Smoking or COPD										
Benedetto, 2014 ^19^	UK	Observational (PSM)	IMA	750/750	669 (89.2)/673 (89.7)	22–50: 14.4%/14.3% 50–60: 33.6%/33.3% 60–70 35.7%/31.2% 70–80: 14.5%/18.7% 80–92: 1.7%/2.5%	119 (15.9)/115 (15.3)	90 (12)/90 (12)	99 (13.2)/104 (13.9)	538 (71.7)/544 (72.5)	MAG	2.88/2.74	N/A	N/A	0.61 (0.38–0.97)	10	4.8	8
Buxton, 2014 ^20^	Australia	Observational (PSM)	RA	384/384	292 (76)/302 (79)	70.3 **±** 8.6/69.7 **±** 8.7	104 (27)/109 (28)	16 (4)/16 (4)	108 (28)/121 (31)	0	TAR	3.4 **±** 0.8/3.4 **±** 0.7	78.1 **±** 23.6/74.9 **±** 23.5	99.6 **±** 26.9/ 101.5 **±** 33.7	0.79 (0.70–0.90)	15	10	8
Dalen, 2014 ^21^	Sweden	Observational (PSM)	IMA	558/558	74.2%/71.9%	64.4/64.5	13.7%/18.4%	4.3%/3.0%	29.7%/27.9%	18.5/22.4	MAG	3.1/3.2	N/A	N/A	1.20 (0.85–1.69)	14	7.5	7
Grau, 2015 ^22^	USA	Observational (PSM)	ITA	1006/1006	901 (89.6)/884 (87.9)	60 **±** 9/62 **±** 9	111 (11.0)/134 (13.3)	51 (5.1)/60 (5.9)	51 **±** 11/50 **±** 12	41 (11.6)/35 (9.9)	MAG	3.5 ± 1/3.5 ± 1	N/A	N/A	0.707 (0.536–0.932)	15	10.5	8
Petrovic, 2015 ^23^	Serbia	Observational (PSM)	RA	100/100	73/73	56.3 ± 6.1/57.1 ± 6.5	39%/43%	9%/8%	48.8 ± 10.7/48.0 ± 10.8	N/A	MAG	3.08 ± 0.66/3.14 ± 0.66	N/A	N/A	0.72 (0.56–0.92)	8	8	8
Tatoulis, 2015 ^24^	Australia	Observational (PSM)	RA	6232/6232	79.2%/78.8%	64.4 ± 10.1/64.7 ± 10.3	32.4%/31.6%	11.3%/12.2%	46.7%/47.6%	8.6%	TAR	3.0 ± 1.1/3.3 ± 1.0	60.6 ± 34.4/63.8 ± 33.6	80.2 ± 43.6/90.7 ± 42.0	0.80 (0.71–0.90)	10	4.9	7
Tranbaugh, 2015 ^25^	USA	Observational (PSM)	RA	1023/1023	75.6%/76.1%	63.1 ± 7.2/62.1 ± 8.8	39.3%/42%	22.2%/21.6%	47.63 ± 11.8/47.8 ± 13.2	0	MAG	3.8 ± 0.9/3.6 ± 0.9	69.6 ± 21.2/62.0 ± 27.1	92.0 ± 24.8/84.3 ± 34.8	0.67 (0.57–0.77)	10	9	7
Locker, 2016 ^26^	USA	Observational (PSM)	ITA	801/801	684 (85.4)/114 (85.8)	59.3 (8.9)/59.3 (10.2)	19.9%/21.3%	7.2%/7.4%	25 (3,1)/27 (3.4)	N/A	MAG	N/A	N/A	N/A	0.763 (0.641–0.909)	15	7.9	7
Schwann, 2016 ^27^	USA	Observational (PSM)	RA	551/551	479 (87)/454 (82)	58.4 ± 10.2/60.6 ± 10.3	100 (18)/94 (17)	46 (8.3)/39 (7.1)	52 ± 10/54 ± 10	96%/95%	MAG	3.5 ± 0.9/3.3 ± 0.8	N/A	N/A	0.763 (0.591–0.990)	16	10	8
Bisleri, 2017 ^28^	Italy	Observational (PSM)	ITA	175/175	128 (73.2)/126 (72)	76 **±** 4/76 **±** 5	82 (46.8)/87 (49.7)	24 (13.7)/24 (13.7)	55 (31.4)/50 (28.4)	36.9%/20.6%	TAR	2.53 ± 0.46/2.6 ± 0.41	44.7 ± 14/45.8 ± 16	65.6 **±** 22/84.5 **±** 31	0.69 (0.46–1,03)	10	7.42	7
Pevni, 2017 ^29^	Israel	Observational (PSM)	ITA	490/490	354 (72.2)/339 (69.2)	≥70: 215 (43.9)/224 (45.7)	490 (100)/490 (100)	46 (4.8)/58 (10.3)	73 (7.6)/66 (11.7)	212 (22.0)/138 (24.4)	MAG	≥3: 360 (73.5)/320 (65.3)	N/A	N/A	0.729 (0.551–0.964)	10	12.2	8
Pu, 2017 ^30^	Canada	Observational (PSM)	RA	5580/5351	89.5%/89.4%	60.0 (8.7)/60.3 (8.5)	29.6%/29.5%	14.6%/14.7%	39.3%/38.9%	4.6%/4.3%	MAG	3.9/3.9	82.5 (29.8)/79 (25.9)	N/A	0.79 (0.72–0.87)	15	9.1	8
DeSimone, 2018 ^31^	UK	Retrospective (PSM)	ITA	1297/1297	18%/19%	<60 y: 58%/56% 60–69 y: 26%/26% 70–79 y: 14%/15% ≥80 y: 3%/2%	16%/17%	8%/9%	24%/25%	9.6/9.6	MAG	2/2	63.6/53.3	93.3/85.1	0.79 (0.69–0.91)	15	12	7
Gatti, 2018 ^32^	Italy	Observational (PSM)	ITA	247/247	0/0	71.7 ± 7.4/ 71.7 ± 7.4	35.2%/36.4%	5.3%/5.3%	78 (31.6)/81 (32.8)	N/A	MAG	N/A	N/A	N/A	0.71 (0.55–0.91)	15	8	8
Iribarne, 2018 ^33^	UK	Observational (PSM)	IMA	1297/1297	75.6%/72.3%	<60: 47.4%/42.4% 60–69: 28.6%/31.3% 70–79: 21.1%/24.9% ≥80: 2.8%/1.4%	100%/100%	11.3%/12.0%	33.8%/27.2%	9.9%/9.7%	MAG	1–1.9/1–1.9	64.3/55.6	94/90	0.75 (0.57–0.98)	15	9.3	8
Rocha, 2018 ^4^	Canada	Observational (PSM)	RA	8629/8629	83.3%/83.5%	63.9 ± 10.0/63.8 ± 9.9	3150 (36.5)/3110 (36)	6.1%/6.2%	38.3%/38.2%	21.2%/20.6%	MAG	3.8 ± 0.8/3.5 ± 0.7	N/A	N/A	0.80 (0.73–0.88)	8	4.2	8
Royse, 2018 ^34^	Australia	Observational (PSM)	RA	232/232	182 (78)/182 (78)	67.0 ± 9.7/67.7 ± 9.8	40 (17)/38 (16)	27 (12)/27 (12)	N/A	N/A	MAG	3.3 ± 0.8/3.3 ± 0.8	N/A	N/A	0.77 (0.625–1.0)	21	7	7
Schwann, 2018* ^35^	USA	Observational (PSM)	RA	3175/3175	46.5%/44.4%	61.96 (9.50)/61.98 (9.76)	37.6%/37.6%	39.2%/38.7%	48.6 (11)/48.6(12)	N/A	MAG	N/A	N/A	N/A	0.83 (0.76–0.92)	15	8.7	7
Chikwe, 2019 ^36^	USA	Observational (PSM)	ITA/RA	3588/3588	84.8%/85%	60.9 ± 10.4/61.1 ± 10.2	40.6%/40.3%	21.7%/21.9%	50.9%/52.1%	49.8%/50.6%	MAG	3.6 ± 1.0/3.6 ± 1.0	N/A	N/A	0.80 (0.72–0.90)	10	7.8	8
Kunihara, 2019 ^37^	Japan	Observational (PSM)	RA	104/104	75 (72)/72 (69)	64 **±** 8/65 **±** 7	104 (100)/104 (100)	60 (58)/56 (54)	58 **±** 16/61 **±** 16	0	TAR	4.3 **±** 1.0/4.1 **±** 0.8	57 **±** 17/53 **±** 20	78 **±** 23/93 **±** 32	0.89 (0.58–1.37)	12	11.2	8
Pevni, 2019 ^38^	Israel	Observational (PSM)	RITA	491/491	0	68.9 ± 9.5/69.5 ± 9.2	201 (45)/217 (44.2)	27 (5.5)/35 (7.1)	73 (14.9)/77 (15.7)	N/A	MAG	N/A	N/A	N/A	0.757 (0.653–0.877)	20	15.6	8
Samadashvili, 2019 ^39^	USA	Observational (PSM)	ITA/RA	10,828/10,828	82.9%/82,2%	61.8 ± 9.6/61.7 ± 9.6	3430 (31.7)/3406 (31.5)	1615 (14.9)/1676 (15.5)	30.7%/30.7%	17.7%/17.7%	MAG	3/3	N/A	N/A	0.86 (0.79–0.93)	7	6.3	7
Taggart, 2019 ^40^	Australia, Austria, Brazil, India, Italy, Poland, and United Kingdom	RCT (ITT)	ITA	1548/1554	1318 (85.1)/1338 (86.1)	63.7 ± 8.7/63.5 ± 9.1	1177 (76.0)/1191 (76.6)	1071 (69.2)/1112 (71.6)	110 (30.6)/117 (30.9)	132 (20.6)/135 (21.8)	MAG	3/3	N/A	N/A	0.96 (0.82–1.12)	10	10	4*
Alsaleh, 2023 ^41^	USA	Observational (PSM)	ITA	2882/2882	79.18%/77.20%	61.86 ± 10.24/63.04 ± 9.83	100%/100%	17.07/18.67	56.59%/57.6%	57.26%/60.13%	MAG	3/3	N/A	N/A	0.75 (0.68–0.83)	15	6.8	8
Jameie, 2024 ^42^	Iran	Observational (PSM)	IMA	986/22,812	92%/73.1%	56.69 ± 9.22/65.47 ± 9.81	287 (29.2)/9270 (40.6)	24 (2.4)/855 (3.8)	0.4872 ± 0.0893/0.4721 ± 0.0945	68 (7.0)/1521 (6.7)	MAG	4 (3–4)/4 (3–4)	45 (39–51)	N/A	0.90 (0.67–1.22)	10	9.23	8
Ren, 2024 ^43^	Australia	Observational (PSM)	IMA	29,711/24,564	81.5%/78.8%	65.3 ± 10/67.1 ± 10.2	34%/39.7%	10.8%/13.6%	16%/20.8%	8%/4.3%	MAG	3.5 ± 1/3.2 ± 0.9	N/A	N/A	0.82 (0.78–0.87)	12	4.9	8
Sabik, 2024 ^44^	USA	Observational (IPW)	ITA/RA	100,404/100,404	84.50/84.10	60.3 ± 10.1/60.2 ± 10.3	37.6%/37.1%	16.5%/16.8%	30.3%/40.6%	N/A	MAG	3.6 ± 1.0/3.5 ± 0.9	76 ± 30/68 ± 28	102 ± 37/94 ± 35	0.86 (0.83–0.89)	12	5.3	8

*Notes:*

* Study quality used Jadad scale: IPW: inverse probability weighting; ITT: intention-to-treat analysis; PSM: propensity-score matching; CI: confidence interval. Some of the data were presented in mean ± SD or directly served as percentages.

* Baseline characteristics were presented for cohorts: Taggart, 2022 MAG: Diabetics vs Non-Diabetics; Schwann, 2018: Diabetics vs. Non-Diabetics.

**Table 3.** Subgroup analyses and meta-regression.

Covariates	Subgroups	Number of studies	HR (95% CI)		Heterogeneity		Subgroup analysis		Univariable meta-regression^a^			Multivariable meta-regression^b^			Overall publication bias analysis	
			Fixed-effects sized	Random-effects sized	*I^2^*	*p*-value	Q_w_ moderator	p-value	Coefficient estimate	SE	*p*-value	Coefficient estimate	SE	*p*-value	Egger's regression test, p-value	Begg and Mazumdar's rank correlation test, *p*-value
Overall		27	0.82 (0.80–0.84)	0.81 (0.78–0.83)	31%	0.07									0.137	0.144
Age	<70	19	0.83 (0.81–0.85)	0.81 (0.79–0.84)	41%	0.03	32.27	0.1503	−0.2804	0.0398	<0.0001***	0.1016	0.0433	0.0188*		
	≥70	8	0.76 (0.70–0.81)	0.76 (0.70–0.81)	0	0.97										
Main second conduits used																
ITA	Yes	17	0.83 (0.81–0.85)	0.81 (0.78–0.85)	40%	0.05	32.89	0.1336	−0.2002	0.0183	<0.0001***	−0.0046	0.0331	0.8893		
	No	10	0.79 (0.76–0.82)	0.79 (0.76–0.82)	0	0.71										
RA	Yes	13	0.83 (0.81–0.85)	0.81 (0.79–0.84)	28%	0.16	35.18	0.0849	−0.2275	0.0255	<0.0001***					
	No	14	0.80 (0.77–0.83)	0.80 (0.75–0.84)	30%	0.14										
Types of revascularization	TAR	7	0.85 (0.82–0.87)	0.85 (0.82–0.87)	0	0.53	29.44	0.2461	−0.2311	0.0174	<0.0001***	0.0721	0.0298	0.0156*		
	MAG	20	0.80 (0.78–0.82)	0.79 (0.76–0.82)	22%	0.19										
Female	No	18	0.81 (0.79–0.83)	0.81 (0.79–0.83)	0%	0.48	34.29	0.1017	−0.1947	0.0243	<0.0001***	−0.0001	0.0007	0.9826		
	Yes	9	0.85 (0.81–0.89)	0.85 (0.80–0.90)	13%	0.33										
Diabetes mellitus (DM)	No	23	0.83 (0.81–0.84)	0.81 (0.79–0.84)	33%	0.07	36.58	0.063	−0.2041	0.0338	<0.0001***	0.0003	0.0007	0.6942		
	Yes	4	0.75 (0.69–0.82)	0.75 (0.69–0.82)	0	0.89										
Low ejection fraction (EF)	≥50%	20	0.83 (0.81–0.85)	0.81 (0.79–0.84)	29%	0.11	35.80	0.074	−0.1886	0.0288	<0.0001***	−0.0016	0.0007	0.0259*		
	<50%	7	0.79 (0.75–0.84)	0.79 (0.72–0.86)	48%	0.06										

*Notes:*

^a^ Value presented for the intercept in univariable analysis

^b^ Value presented for each covariates in multivariable analysis

*p*-value of meta-regression would be significant if < 0.05: *** *p* < 0.001: Highly significant; ** *p* < 0.01: Very significant; * *p* < 0.05: Significant; *p* > 0.05: Not significant.

NOS: Newcastle–Ottawa Scale; CI: confidence interval.

### Primary endpoint

#### Overall mortality

A meta-analysis of long-term mortality involving 27 studies, as shown in Figure 3, reveals an overall HR of 0.81 (95% CI, 0.78–0.83; *p* < 0.01), indicating that the use of MAG reduces long-term mortality by approximately 19% compared to the use of SAG. A funnel plot for the associated outcome was constructed for publication bias assessment, as shown in Figure 4. The results of both Egger's regression (*p* = 0.137) and Begg and Mazumdar's test (*p* = 0.144) indicated no significant effect.

#### Subgroup analysis and meta-regression analysis

Subgroup analysis and meta-regression analysis involving 27 studies with specific covariates of interest associated with long-term mortality were performed with the regression method, as shown in Table 3. In all subgroups, composed of age, main second conduits, types of revascularization, gender, DM status, and EF status, the long-term mortality rate was overall significantly lower in MAG compared to SAG, with various levels of heterogeneity. No differences were found between subgroups, supported further by the insignificant Q_w_ value, which suggests that the results of MAG use have no significantly different effects within subgroups. However, the distribution of the studies between subgroups was not evenly assigned, meaning that the analysis itself might not be capable of detecting differences.

In univariable meta-regression, all covariates were found to be associated with long-term mortality, as shown in Table 3. After adjustments for all covariates, the modified effect in multivariable analysis, there are several associations found between some covariates and long-term mortality, implying that the risk of decreased long-term survival in the use of MAG compared to SAG might be predicted with the increasing age, the use of the TAR method, and the reduced EF. Moreover, the absence of statistically significant differences between specific subgroups should be interpreted cautiously, as the unequal distribution of studies and lack of individual patient-level data may have limited the statistical power to detect actual effects.

## Discussion

Our meta-analysis, comprising 27 studies (26 observational and one randomized trial), confirms that the use of multiple arterial grafts in CABG is associated with significantly improved long-term survival compared to the use of a single arterial graft. Although minor variations in the definitions of MAG and TAR existed among included studies, we applied standardized criteria to harmonize these definitions. Our sensitivity analysis demonstrated that excluding studies with broader definitions did not significantly alter the pooled results, suggesting that the impact of this variation on the final outcomes was minimal.

Our analysis demonstrates that MAG reduces long-term mortality by approximately 20% compared to SAG, a clinically significant benefit. This finding aligns with evidence from large observational cohorts. For example, a recent analysis of more than one million CABG patients found that MAG recipients had better long-term survival than those who received SAG.^44^ Such data reinforce the survival advantage of multiple arterial revascularization. However, these results do contrast with some prior reports. A 2020 meta-analysis found no significant difference in long-term mortality between MAG and SAG.^45^ Moreover, the only large-scale randomized trial to date—the Arterial Revascularization Trial (ART)—reported no significant difference in 10-year survival between bilateral-ITA grafting and single-ITA grafting.^46,47^ These discrepancies between observational studies and randomized trials have been noted by other investigators.^47,48^ One explanation is that patients selected for MAG in practice tend to be younger and healthier, whereas the randomized trial setting equalizes patient factors, potentially diluting the apparent benefit of MAG. Unmeasured confounders and crossover of graft strategies in trials (as occurred in ART) can obscure actual differences in outcomes. Our meta-analysis synthesizes a broad range of evidence and suggests that, when taken together, the data do support a survival benefit from multiple arterial grafting, even though RCTs have so far been inconclusive.

Several factors likely explain the apparent discrepancy between our findings and the neutral results of the ART trial. First, the interventions compared were not identical. Arterial Revascularization Trial focused on BITA versus single internal thoracic artery (SITA) grafting, whereas our meta-analysis evaluated MAG, which frequently included a RA in addition to one or two ITAs. Thus, the biological contrast between groups in ART was narrower. Second, there was substantial treatment crossover: approximately 14% of patients in the BITA group ultimately received only one ITA, while nearly 22% of patients in the SITA group also received a second arterial conduit, most often a RA. This contamination likely diluted any survival difference. Third, outcomes in both groups of ART were excellent, reflecting a highly controlled trial environment with experienced surgeons and rigorous perioperative care, which may have potentially masked benefits observable in broader real-world practice. Lastly, the trial was underpowered due to lower-than-expected event rates, making it challenging to detect modest but clinically meaningful survival differences. Notably, post hoc as-treated analyses of ART suggested a trend favoring patients who received two or more arterial grafts, consistent with the direction of our pooled findings.

Notably, the survival benefit of MAG was observed across all examined subgroups in our analysis. In both younger patients and those aged 70 or older, MAG was associated with lower long-term mortality than SAG. This is consistent with recent research showing that even elderly patients can derive a survival advantage from multiple arterial grafts.^11^ The likely reason is the superior longevity of arterial conduits: arterial grafts (ITA or RA) have a much lower risk of late occlusion compared to vein grafts, which translates into more sustained myocardial perfusion and fewer cardiac events over time. Indeed, MAG has been associated with reduced incidence of late myocardial infarction and recurrent angina, owing to better graft patency. Nonetheless, it is worth noting that some experts caution against indiscriminate use of MAG in certain high-risk elderly patients. Patients over 70 with significant comorbidities (yielding limited life expectancy), or those with small, diffusely diseased target vessels or less critical coronary stenoses, may not experience the same magnitude of benefit from multiple arterial grafts.^49,50^ In such cases, the incremental surgical risk (e.g., a higher risk of sternal wound complications with BITA harvest) might outweigh the long-term benefit. These considerations underscore the importance of individualized patient selection, even as we advocate for the broader use of MAG.

Regarding the choice of the second conduit in MAG, our findings indicate that whether a second ITA (RITA) or the RA is used, long-term survival is significantly better than with a single arterial (LITA) graft alone. This underscores the importance of having a second arterial graft rather than a vein. In practice, the decision between RITA and RA often depends on patient factors. For instance, BITA grafting may be avoided in patients with obesity or poorly controlled diabetes due to the elevated risk of deep sternal wound infection, whereas using the RA requires adequate ulnar collateral circulation to tolerate radial harvest. Current evidence tends to favor the RA as the second arterial conduit when both options are available.^10,51^ Comparative studies have shown that RA grafts provide similar long-term survival rates as RITA grafts at mid-term follow-up; however, RITA use carries a higher risk of sternal wound complications and slightly higher early mortality in some analyses.^51^ Thus, from a clinical standpoint, a LITA + RA strategy is often considered the safest way to achieve multiple arterial revascularization in suitable patients, reserving BITA usage for those at lower risk of sternal complications.

We also evaluated outcomes based on the total number of arterial grafts. Both MAG (typically two arterial grafts with additional vein grafts as needed) and TAR (using only arterial grafts) were associated with better long-term survival compared to the traditional LITA + vein approach. Notably, TAR is effectively an intensified form of MAG. Some evidence suggests that TAR might confer the most significant benefit; for example, one large meta-analysis observed a trend toward improved survival in TAR over MAG (although not statistically significant).^52^ Similarly, a post hoc analysis of the ART trial data indicated a stepwise improvement in outcomes moving from SAG to MAG to TAR.^53,54^ These observations imply that the more arterial conduits utilized, the better the long-term outcome might likely reflect the avoidance of SVGs altogether. However, fully arterial revascularization can be technically demanding and may not be feasible or necessary for all patients. Our results support the idea that any increase in arterial graft use is beneficial; achieving TAR might offer incremental advantages, but even using two arterial grafts (MAG) versus one is a significant improvement.

Gender-specific effects were also noted. Male patients in our analysis appeared to gain more pronounced survival benefit from MAG compared to female patients. In women, the advantage of MAG was less clear, which could be due to several factors. Women generally have smaller coronary artery diameters and more diffuse disease, which might reduce graft patency rates and lessen the impact of multiple arterial conduits. There may also be differences in comorbidities and hormonal factors that influence outcomes.^55,56^ It is possible that the benefit of MAG in women, although still present, is more modest or requires a more extended follow-up period to become evident. However, in the present analysis, sex-specific data regarding conduit selection were inconsistently reported across studies. Consequently, we were unable to perform a robust comparison of RITA versus RA utilization or outcomes specifically among women. Hence, further research is needed to determine whether there is a true interaction between patient sex and the efficacy of multiple arterial grafting, or whether other variables confound our observation, given the unclear magnitude of the observed benefit among women. This should incorporate sex-specific analyses or female-focused registries to clarify whether differences in conduit selection, anatomical characteristics, or the historical underrepresentation of women in surgical studies contribute to these uncertain outcomes.

Left ventricular function is another important consideration. We found that in patients with moderately reduced EF (e.g., EF 35–50%), multiple arterial grafting was associated with better survival than SAG. Arterial grafts may provide improved perfusion to hibernating myocardium, which is particularly valuable in the setting of impaired ventricular function. However, in patients with severely depressed EF (<35%), some studies have reported no significant outcome difference between MAG and SAG.^57^ When EF is very low, the overall prognosis is dominated by poor ventricular function, and the relative benefit of an additional arterial graft may be attenuated. Additionally, surgeons might select only the most critical targets for grafting in such high-risk patients, limiting the opportunity for MAG to make a difference. Our subgroup findings suggest that while MAG is generally advantageous, its absolute benefit might be smaller in the sickest heart failure patients, and surgical teams should weigh the complexity of multiarterial procedures against the patient's frailty in this context.

We performed meta-regression analyses to explore whether specific patient or study characteristics might influence the observed mortality benefit. The results indicated that increasing patient age, use of a total arterial approach, and lower EF were associated with some variation in long-term mortality outcomes. In particular, the survival advantage of MAG tended to diminish slightly with very advanced age or with severely reduced EF, as discussed above. Interestingly, our meta-regression also noted that studies with a higher proportion of TAR use showed some differences, potentially reflecting the selection of healthier patients for TAR. Taggart et al. likewise found that age and diabetes were significant factors affecting long-term outcomes in their secondary analysis of the ART trial.^54^ Overall, none of these factors eliminated the benefit of MAG, but they underscore that patient characteristics can modify the degree of benefit. This highlights the importance of tailoring the revascularization strategy to the individual. It also highlights the need for further prospective research, ideally involving additional randomized trials or advanced observational analyses, to confirm the benefit of MAG in specific subpopulations and to address the gaps left by prior trials.

## Limitations

Our analysis was explicitly designed to compare MAG with SAG. Although it included cases TAR, the available data were not uniformly reported in a manner that allowed for a reliable distinction between TAR and other MAG configurations. Determining the optimal second arterial conduit remains a key unresolved clinical question. While the RA is often preferred because of its ease of harvest and lower risk of sternal wound complications, the RITA may offer unique long-term survival advantages. Unfortunately, our aggregate-level data did not permit a direct head-to-head comparison between RA and RITA. Future patient-level meta-analyses and RCTs will be essential to clarify whether TAR provides additional benefits and to determine whether RITA or RA is the superior second conduit.

There are several more essential limitations to our study. Firstly, only one randomized trial was included in the analysis. Although we used adjusted effect estimates (HRs) and performed subgroup and meta-regression analyses to account for confounding, the possibility of residual bias remains. While most included studies adjusted for baseline patient characteristics using advanced statistical methods, unmeasured confounders such as surgeon experience and institutional procedural volume were not consistently reported and could not be accounted for in our pooled analysis. Patients chosen for multiple arterial grafts may differ systematically from those receiving a single arterial graft, as surgeons often select younger or healthier patients for MAG. Although most included studies used robust statistical adjustments such as PSM or IPW, unmeasured factors and residual selection bias cannot be completely ruled out. Moreover, the subgroup and meta-regression analyses were also further limited by the unequal distribution of studies across categories and the absence of individual patient-level data. As a result, these analyses may have been underpowered, and nonsignificant findings should not be interpreted as evidence of no actual difference. Future patient-level meta-analyses are needed to clarify subgroup effects more definitively.

Secondly, we included a broad range of patient populations and surgical practices. We did not restrict inclusion based on patient age or comorbidities, and we did not require that the second arterial graft be used on a specific target (e.g., the LAD) or any use of an additional arterial conduit qualified as MAG. This inclusive approach not only improves generalizability but it also introduces heterogeneity. The studies varied in their definitions of MAG (some included TAR, others allowed one artery + one vein as MAG), and surgical techniques and patient management spanned several decades. Lastly, our analysis could not account for all granular factors, such as surgeon experience or the use of complementary strategies such as off-pump surgery or enhanced medical therapy, which may impact long-term outcomes. Moreover, despite a large cumulative sample size, our meta-analysis is only as robust as the data available. Key outcomes, such as graft patency and nonfatal events, were not uniformly reported, and long-term mortality was the primary focus. In summary, caution is warranted when interpreting our results, given the above limitations; however, they align with a substantial body of evidence that favors multiple arterial grafting.

## Conclusion

In conclusion, our systematic review and meta-analysis provide strong evidence that multiple arterial grafting in CABG offers a long-term survival advantage over the conventional single arterial (LITA) plus vein graft strategy. The mortality benefit of MAG was consistent across diverse patient groups, supporting the broader adoption of multiple arterial conduits to improve outcomes in coronary surgery. These findings lend support to current guidelines and expert recommendations that encourage the use of a second (or third) arterial graft when feasible. However, the choice of revascularization strategy should always be individualized to balance potential survival gains against operative complexity and patient-specific risk profiles. Surgeons must consider patient-specific factors, such as age, comorbid conditions, anatomical suitability, and operative risk, when deciding on MAG, as not every patient will be an ideal candidate for multiple arterial grafts. For example, in clinical practice, the long-term survival advantage of multiple arterial grafting appears most pronounced among younger patients, those with preserved ventricular function, and individuals with fewer comorbidities. Conversely, in elderly patients with severe comorbidities or markedly reduced left ventricular function, the incremental benefit may be attenuated due to higher perioperative risk and limited life expectancy. Ultimately, our study reinforces the clinical importance of optimizing graft selection in CABG and highlights the need for continued research (including large-scale randomized trials) to define further the role of multiple arterial grafting in improving long-term survival for patients with CAD.
